# Noninvasive ventilation with helium–oxygen mixture in hypercapnic COPD exacerbation: aggregate meta-analysis of randomized controlled trials

**DOI:** 10.1186/s13613-017-0273-6

**Published:** 2017-06-06

**Authors:** Fekri Abroug, Lamia Ouanes-Besbes, Zeineb Hammouda, Saoussen Benabidallah, Fahmi Dachraoui, Islem Ouanes, Philippe Jolliet

**Affiliations:** 1 Intensive Care Unit, CHU Fatouma Bourguiba, Research Laboratory LR12SP15, University of Monastir, 5000 Monastir, Tunisia; 2 Département des Centres Interdisciplinaires et de Logistique Médicale, Lausanne, Switzerland

**Keywords:** COPD, Exacerbation, Acute respiratory failure, Noninvasive ventilation, Helium

## Abstract

When used as a driving gas during NIV in hypercapnic COPD exacerbation, a helium–oxygen (He/O_2_) mixture reduces the work of breathing and gas trapping. The potential for He/O_2_ to reduce the rate of NIV failure leading to intubation and invasive mechanical ventilation has been evaluated in several RCTs. The goal of this meta-analysis is to assess the effect of NIV driven by He/O_2_ compared to air/O_2_ on patient-centered outcomes in hypercapnic COPD exacerbation. Relevant RCTs were searched using standard procedures. The main endpoint was the rate of NIV failure. The effect size was computed by a fixed-effect model, and estimated as odds ratio (OR) with 95% confidence interval (CI). Additional endpoints were ICU mortality, NIV-related side effects, and the length and costs of ICU stay. Three RCTs fulfilled the selection criteria and enrolled a total of 772 patients (386 patients received He/O_2_ and 386 received air/O_2_). Pooled analysis showed no difference in the rate of NIV failure when using He/O_2_ mixture compared to air/O_2_: 17 vs 19.7%, respectively; OR 0.84, 95% CI 0.58–1.22; p = 0.36; *I* ^2^ for heterogeneity = 0%, and no publication bias. ICU mortality was also not different: OR 0.8, 95% CI 0.45–1.4; p = 0.43; *I* ^2^ = 5%. However, He/O_2_ was associated with less NIV-related adverse events (OR 0.56, 95% CI 0.4–0.8, p = 0.001), and a shorter length of ICU stay (difference in means = −1.07 day, 95% CI −2.14 to −0.004, p = 0.049). Total hospital costs entailed by hospital stay and NIV gas were not different: difference in means = −279$, 95% CI −2052–1493, p = 0.76. Compared to air/O_2_, He/O_2_ does not reduce the rate of NIV failure in hypercapnic COPD exacerbation. It is, however, associated with a lower incidence of NIV-related adverse events and a shortening of ICU length of stay with no increase in hospital costs.

## Background

Noninvasive ventilation (NIV) has become a standard of care in COPD patients with acute exacerbation requiring ventilatory support [1–4]. Avoiding tracheal intubation drastically reduces the rate of ventilator-associated pneumonia (VAP), antibiotic use, the time spent under mechanical ventilation, ICU length of stay, and associated mortality [5–9]. The sustained mastering of the clinical and technological aspects of NIV (defining optimal indications, selection of ventilators and interface, improvements in patient–ventilator synchrony) has been associated with substantial advances in NIV success rates, allowing a wide range of patients to be managed entirely by this technique, thereby minimizing the risk of complications inherent to conventional invasive ventilation [2, 8, 9]. Despite these advances, it is believed that an additional success margin is possible, leading to further reduction in the number of patients still in need of invasive ventilation. One such area of potential progress is the gas used for ventilation [10–12].

Compared to air–oxygen (air/O_2_), a mixture of helium and oxygen (He/O_2_) has been consistently shown to convey numerous beneficial effects in the setting of increased airway resistance owing to its lower density. Indeed, the lower density of helium enhances the transition from a turbulent to a laminar flow, thereby reducing density-dependent components of airway resistance within bronchi with increased resistance, as is the case in COPD exacerbation [10–16]. These effects translate into a reduction in dynamic hyperinflation and a lower work of breathing [10, 15, 17]. These studies provide sound scientific grounds to anticipate a reduction in NIV failure rate when using He/O_2_ instead of air/O_2_ in COPD exacerbation requiring ventilatory support [10]. This hypothesis has been tested in randomized controlled trials (RCTs).

The aim of the present systematic review and meta-analysis is to compare the effect of He/O_2_ and air/O_2_ NIV on patient-centered clinical outcomes.

## Methods

### Search strategy and study selection

 Relevant studies were searched in MEDLINE, EMBASE, and Science Citation Index with the restriction of randomized clinical trial for article type published up to September 20, 2016, with the following MeSH terms: [“non-invasive ventilation” or “Bilevel”] AND [(“pulmonary disease, chronic obstructive”[MeSH Terms] OR (“pulmonary”[All Fields] AND “disease”[All Fields] AND “chronic”[All Fields] AND “obstructive”[All Fields]) OR “chronic obstructive pulmonary disease”[All Fields] OR “copd”[All Fields]) AND “exacerbation”[All Fields] AND [“heliox” or “helium–oxygen” or “helium”]. We have also conducted a manual search in journals and contacted authors of trials.

### Study selection

We included all randomized controlled clinical trials designed to evaluate the efficacy and safety of NIV using a mixture of helium and oxygen to ventilate COPD patients with acute hypercapnic respiratory failure. Standard treatment (e.g., bronchodilators and antibiotics) had to be comparable in control and intervention arms. Patients included in these studies were adults aged 18 and older with COPD diagnosed on clinical criteria and respiratory function tests.

### Data extraction and study characteristics

Two independent evaluators (FA and LOB) selected studies according to the inclusion criteria and extracted the following: type and baseline characteristics of included patients, the criteria for NIV, type and composition of He/O_2_ mixture (78/22 or 65/35%), time to the first NIV session and its duration, total duration of He/O_2_ administration, minimum NIV duration with a given gas mixture during the first 24 h, composition of the gas administered between NIV sessions (whether helium/O_2_ or air/O_2_), type of associated medications, and criteria for primary and secondary endpoints. Disagreements were resolved by consensus.

Data were extracted to allow quality assessment of the included studies. The risk of bias tool from the Cochrane Handbook was used [18].

### Data synthesis

In this meta-analysis, the primary endpoint was the rate of NIV failure during the index ICU stay. The secondary endpoints included the intubation rate per se as the definition of NIV failure was not uniform; in one study, the failure rate was a composite of necessity of intubation and/or death without intubation during the ICU stay [19]. Additional endpoints were ICU mortality, the length of ICU stay, and the costs of ICU stay. Safety was assessed through the number of serious adverse events related to He/O_2_ mixture, and the number of episodes of complication related to NIV. The latter consisted of facial skin necrosis, gastric distension, pneumothorax, and nosocomial pneumonia. NIV failure was not considered an NIV adverse effect since it was counted separately as the primary outcome.

### Statistical analysis

For binary outcomes (NIV failure rate, intubation rate, mortality, NIV complications, and adverse effects of He/O_2_ mixture), we reported the effect sizes estimates as odds ratios (ORs) with 95% confidence intervals (CIs). For the length of ICU stay, and the difference in costs of the total hospitalization per patient, results were expressed as difference of means and 95% CIs. Only two out of three included studies reported the total costs per patient, which consisted of both the costs of hospital stay and those of the gas used for noninvasive ventilation. The first study was a Swiss one [20], and expressed the expenses in US$, while the second was a multicenter study and reported detailed costs in French patients relying on diagnosis-related group (DRG) tools [19]. In the latter, costs were expressed in euros, and converted to US$ (1€ = 1.1386US$).

Statistical significance was set at p < 0.05 for hypothesis testing and p < 0.1 for heterogeneity testing. We measured heterogeneity and expressed it as *I* ^2^, with suggested thresholds for low (*I* ^2^ = 25–49%), moderate (*I* ^2^ = 50–74%), and high (*I* ^2^ ≥ 75%) values. We used a fixed-effect model which assumes that studies included in the meta-analysis should share a common effect size, since patients’ characteristics and the evaluated intervention are similar in all studies. To assess publication bias, we visually examined the funnel plot for NIV failure and performed the Egger test of the intercept which uses precision to predict the standardized effect. All statistical tests were two-sided.

The meta-analysis was conducted using the Comprehensive Meta-Analysis (CMA) program version 2 software (Biostat, Englewood, NJ, USA). This meta-analysis was conducted in accordance with the PRISMA guidelines.

## Results

### Search results and trials characteristics

**Fig. 1:**
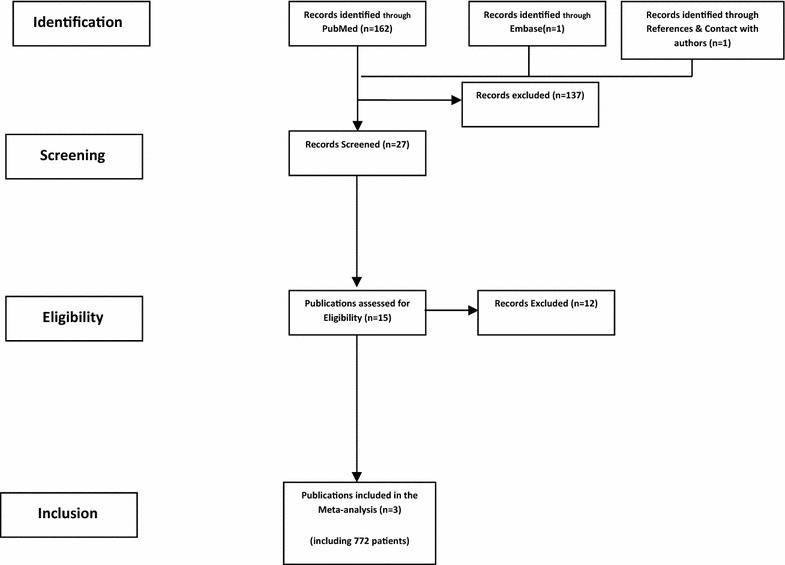
PRISMA diagram of the study selection process

The literature search initially identified 164 citations. Among these studies, only 15 dealt with the use of He/O_2_ for NIV in COPD exacerbation. Of these, three randomized controlled studies evaluating the efficacy of NIV using He/O_2_ in acute COPD exacerbation were included in the final analysis [19–21]. The selection process is illustrated by the flowchart in Fig. 1. The included studies enrolled a total of 772 patients. The main clinical characteristics of included studies are depicted in Table 1.

**Table 1:** Characteristics of included studies

RCT	Sample size		Baseline FEV1 (ml/s)	He/O_2_ mixture	Ventilator type/helium canister connection	Ventilation mode/daily duration/study duration	Interface	Between NIV sessions gas	NIV failure criteria	Baseline pH		Baseline PaCO_2_ (mmHg)		Predicted mortality rate (%)	SMR	Predicted NIV failure rate (%)	Observed NIV failure rate (%)	
	He/O_2_	Air/O_2_								He/O_2_	Air/O_2_	He/O_2_	Air/O_2_				He/O_2_	Air/O_2_
Jolliet_2003	59	64	740 ± 362	78/22	ICU ventilator with connection to the air inlet	NIPSV/≥ 6H/until recovery	Oronasal mask	Air/O_2_	Intubation	7.32 ± 0.06	7.30 ± 0.07	65 ± 13	63 ± 15	24	0.33	45	13.5	20.3
Maggiore_2010	102	102	900 ± 400	65/35	ICU ventilator with connection to the O_2_ inlet	NIPSV/≥ 6H/until recovery	Facial full mask	Air/O_2_	Intubation	7.28 ± 0.07	7.28 ± 0.06	73 ± 18	72 ± 15	15	0.67	40	24.5	30.4
Jolliet_2016	225	220	785 ± 360	78/22	ICU ventilator with dedicated connection	NIPSV/≥ 6H/≤ 72H	Oronasal mask	He/O_2_	Intubation or death in the ICU	7.29 ± 0.05	7.30 ± 0.06	71 ± 16	68 ± 17	15	0.37	25	14.7	14.5

*FEV1* forced expiratory volume in 1 s, *NIPSV* noninvasive pressure support ventilation, *SMR* standardized mortality ratio

#### Quality assessment

The three studies were randomized, controlled, non-blinded studies. The risk of bias regarding random sequence generation and allocation concealment was low in the study by Maggiore et al. [21] and unclear in the remaining two. Blinding of patients was possible in the three studies. All studies were open-label regarding physicians’ assessment of outcomes, which were either hard outcomes such as ICU mortality, or relied on pre-defined objective criteria such as the main efficacy criteria (tracheal intubation). In the most recent study by Jolliet et al. [19], an adjudication and safety committee determined in a blinded manner whether intubation criteria were met in every case. All included studies had low bias for incomplete data. There was no selective outcome reporting bias in the three studies (Table 2).

**Table 2:** Quality assessment of RCTs

Study	Random sequence generation	Allocation concealment	Blinding of patients	Blinding of outcome assessment	Incomplete outcome data		Selective outcome reporting
					He/O_2_ group	Air/O_2_ group	
Jolliet_2003	UNCLEAR Stated only that patients were randomized	UNCLEAR	LOW Patients blind to the type of driving gas	UNCLEAR Hard outcomes such as mortality and pre-defined criteria of intubation	LOW All results are based on all patients (ITT)	LOW All results are based on all patients (ITT)	LOW No apparent selective reporting
Maggiore_2010	LOW Computer-generated randomization	LOW Randomization undertaken at central site with a computer-generated allocation sequence	LOW Patients blind to the type of driving gas	UNCLEAR Hard outcomes such as mortality and pre-defined criteria of intubation	LOW All results are based on all patients (ITT)	LOW All results are based on all patients (ITT)	LOW No apparent selective reporting
Jolliet_2016	UNCLEAR Stated only that eligible patients were randomized	UNCLEAR	LOW Patients blind to the type of driving gas	UNCLEAR Hard outcomes such as mortality and pre-defined criteria of intubation	LOW All results are based on all patients (ITT)	LOW All results are based on all patients (ITT)	LOW No apparent selective reporting

The studies included a majority of males (65%) with a mean age of 69 ± 14 years (Table 1). All studies included COPD patients (mean baseline FEV1 = 808 ± 110 ml), experiencing severe exacerbation requiring ventilatory support. COPD diagnosis was either known or suspected on smoking status, clinical and radiologic signs, and respiratory function tests. The need for ventilatory support and ICU admission relied on the association of respiratory acidosis (pH ≤ 7.35 and PaCO_2_ ≥ 45 mmHg), and a respiratory rate ≥25 b/min. In the group of patients receiving He/O_2_, the gas mixture composition varied among studies with similar formulations: He/O_2_ 78/22% in both studies conducted by Jolliet et al. [19, 20], and a 65/35% formulation in the study by Maggiore et al. [21]. In the most recent study by Jolliet et al., the group of patients allocated to He/O_2_ also received this mixture continuously during the first 72 h after inclusion, both during NIV sessions and during spontaneous breathing between NIV sessions [19]. In the previous studies, patients belonging to both study arms inhaled an air/O_2_ mixture between NIV sessions.

Overall, the severity of the index exacerbation was high as inferred from the baseline arterial pH (7.3 as a mean in two studies and 7.28 in one study) and from the predicted mortality derived from mortality prediction systems (SAPS and APACHE scores): between 15 and 24% in the three studies. NIV failure was defined as the need for tracheal intubation in the studies by Jolliet and Maggiore [20, 21], and by the need for intubation or death in the ICU without intubation, in the ECHO ^ICU^ trial [19].

### Data analysis

Comparison of NIV gas mixtures involved 386 patients who received He/O_2_ and 386 ventilated with air/O_2_.

#### Primary endpoint

**Fig. 2:**
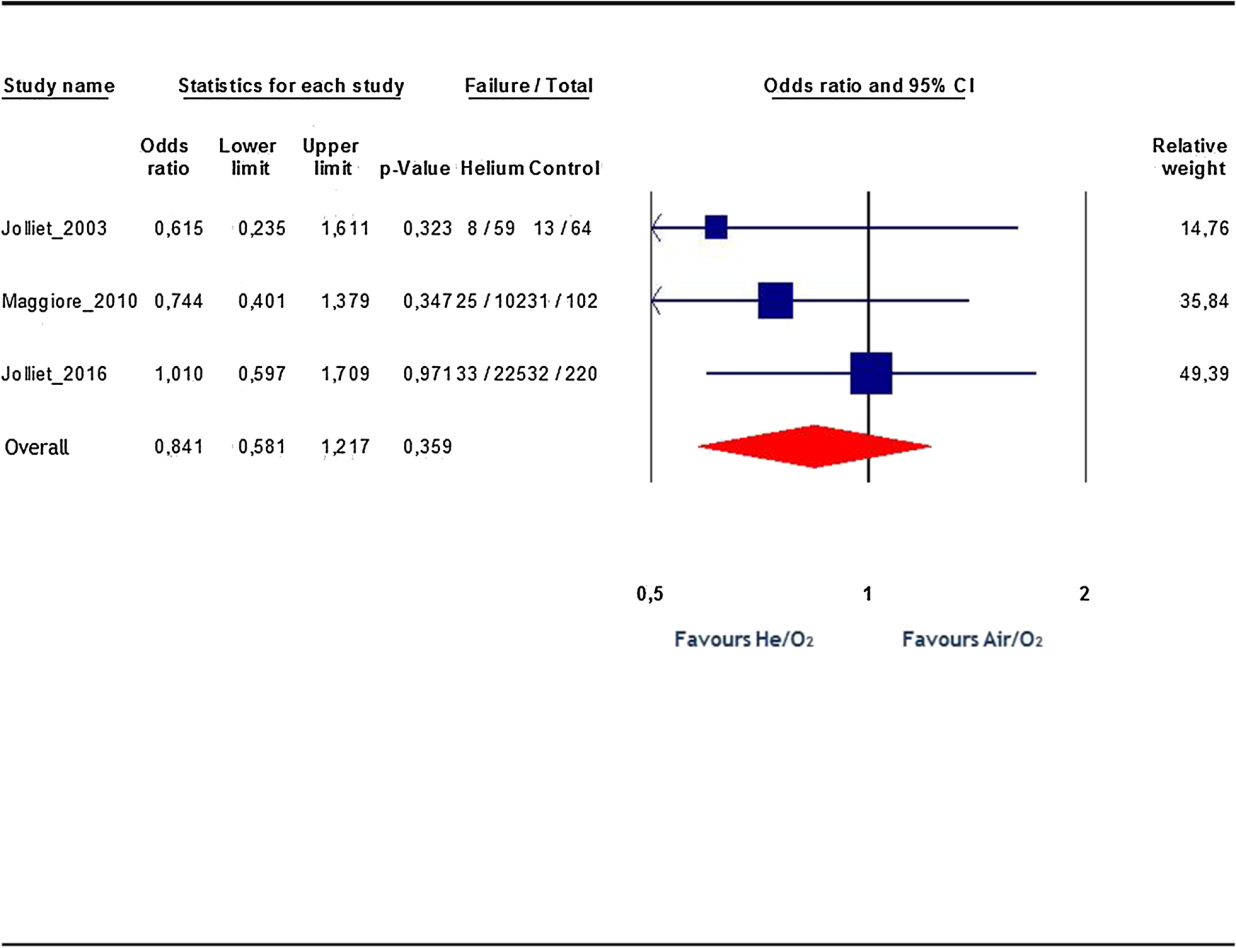
Effects of He/O_2_ mixture on NIV failure rate. Blue squares represent odds ratios (ORs) in individual trials with the size proportional to the weight of the study. The 95% confidence intervals (CIs) for individual trials are denoted by* lines*. The contribution of each included study to the pooled estimate (weight) is plotted as a percentage in the* right column*. The combined overall effect is represented by the* red diamond*

Pooled analysis shows no statistically significant difference in the rate of NIV failure when using He/O_2_ mixture compared to air/O_2_: 17 vs. 19.7%, respectively; OR 0.84, 95% CI 0.58 to 1.22; p = 0.36 (Fig. 2). Overall, there was no heterogeneity (*I* ^2^ = 0%). There was no obvious publication bias detected by visual inspection of the “funnel plot.” The Egger test was also non-significant (regression intercept = −2.18, p = 0.3). We also computed the aggregate effect on the need for tracheal intubation *per se*, as it was a common definition of NIV failure in the included studies. The pooled analysis of the intubation rate reported in the three studies yielded no statistically significant difference between patients ventilated with He/O_2_ or air/O_2_: OR 0.81, 95% CI 0.56–1.17; p = 0.27; *I* ^2^ = 0%.

#### Secondary endpoints

**Fig. 3:**
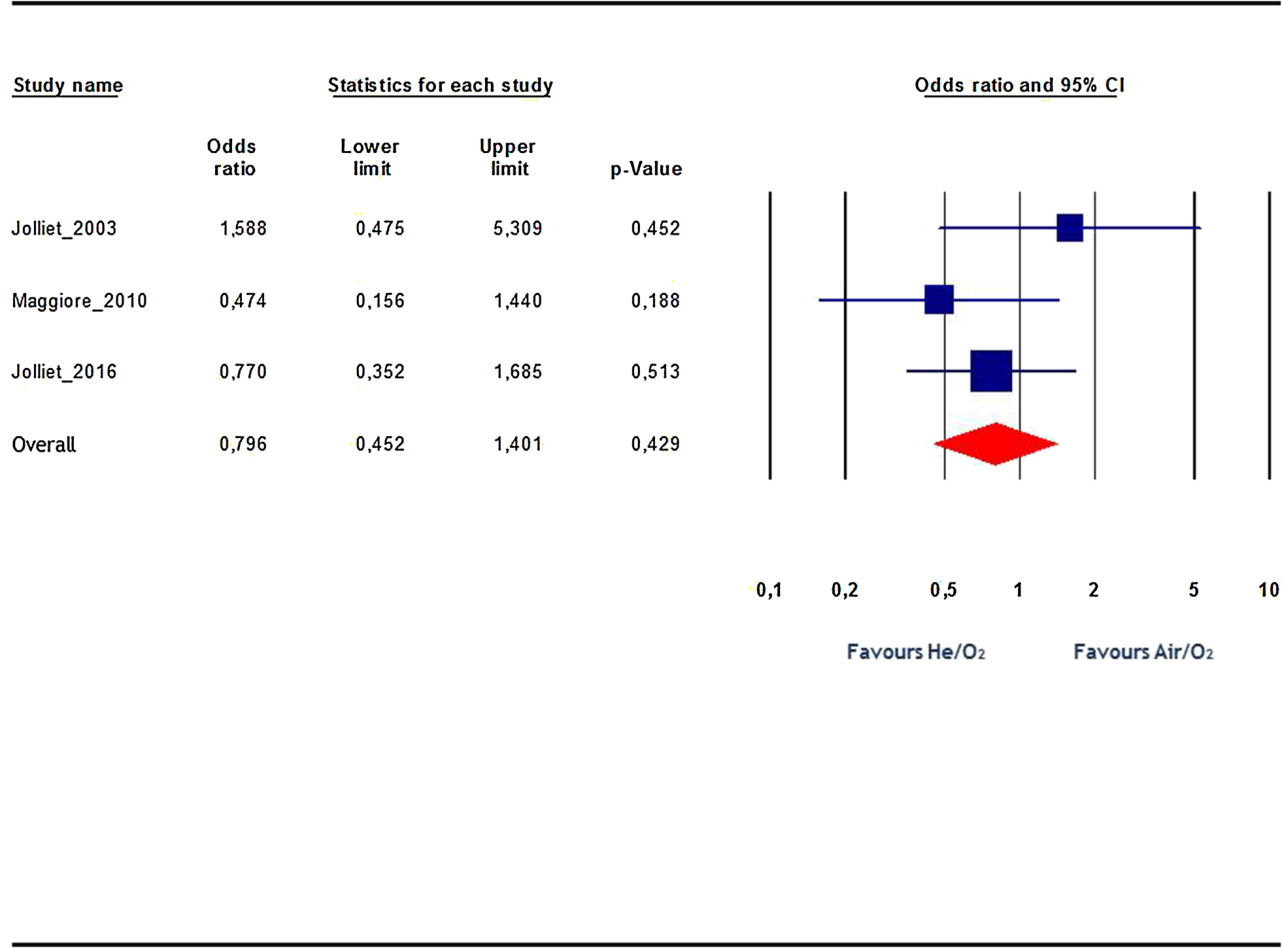
Effects on ICU mortality rate. *Blue squares* represent odds ratios (ORs) in individual trials, while the* red diamond* represents the combined overall effects. I^2^ test for heterogeneity: 5%

Overall, the ICU mortality rate was not statistically different between the He/O_2_ and air/O_2_ groups: OR 0.8, 95% CI 0.45–1.4; p = 0.43; *I* ^2^ = 5% (Fig. 3).

**Fig. 4:**
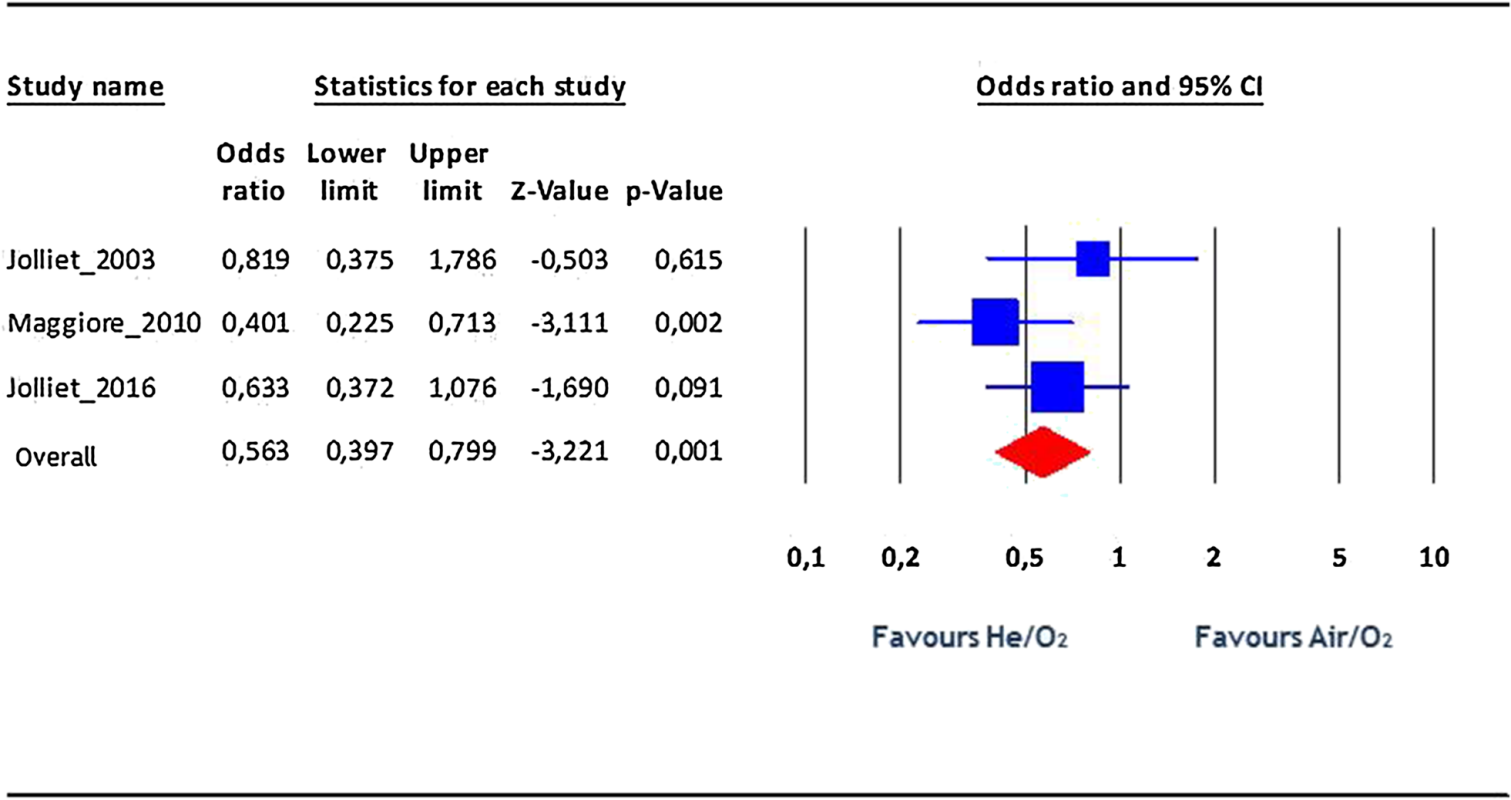
Rate of NIV complications. *Blue squares* represent odds ratios (ORs) in individual trials, while the *red diamond* represents the combined overall effects. I^2^ test for heterogeneity: 0.02%

No adverse event attributable to He/O_2_ was reported. Regarding NIV complications (facial skin necrosis, gastric distension, pneumothorax, and nosocomial pneumonia), there was a statistically significant difference, with less events in the He/O_2_ patients: OR 0.56, 95% CI 0.4–0.8, p = 0.001, *I* ^2^: 0.02 (Fig. 4).

**Fig. 5:**
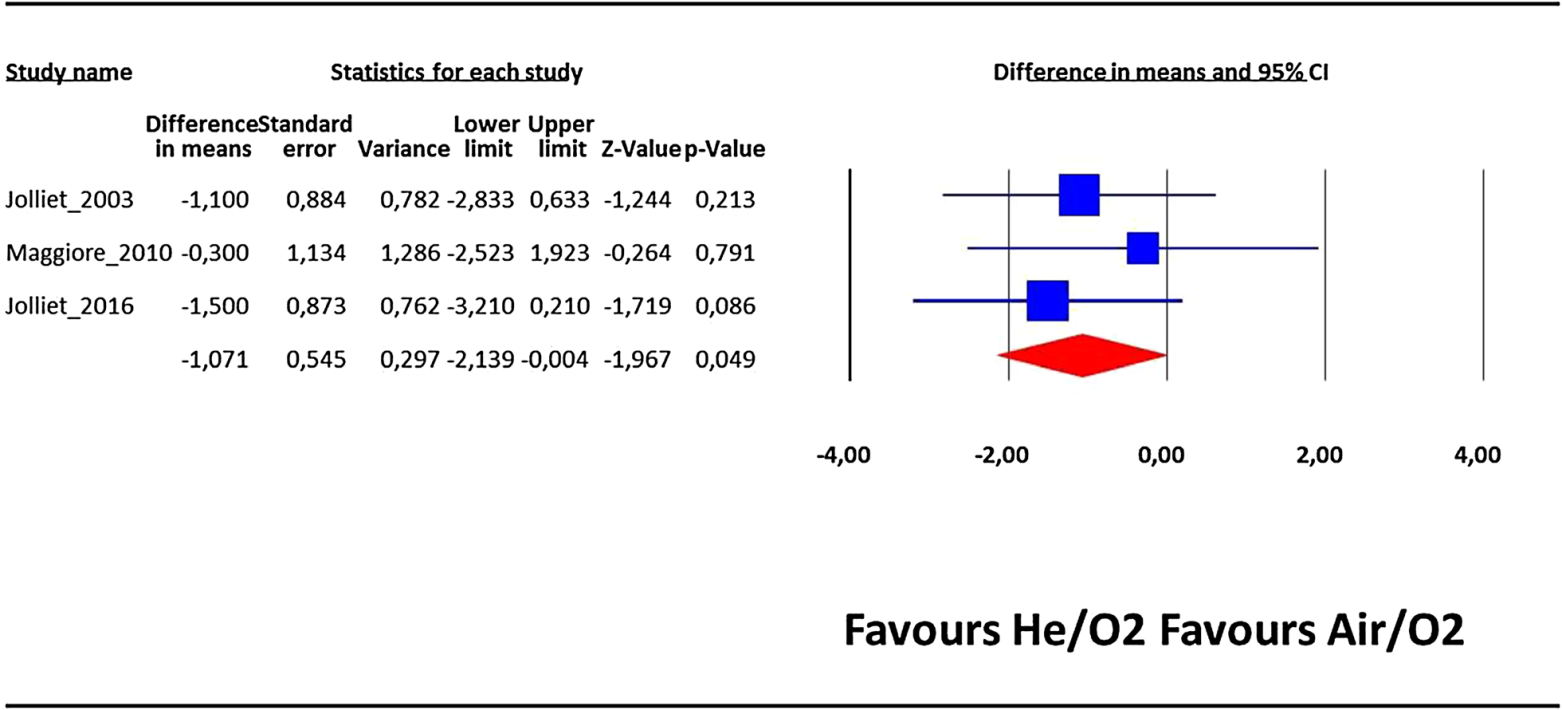
Length of ICU stay. Estimates are expressed as difference in means and 95% confidence. The length of stay was significantly lower in the He/O_2_ with no heterogeneity between included studies (I^2^: 0%)

**Fig. 6:**
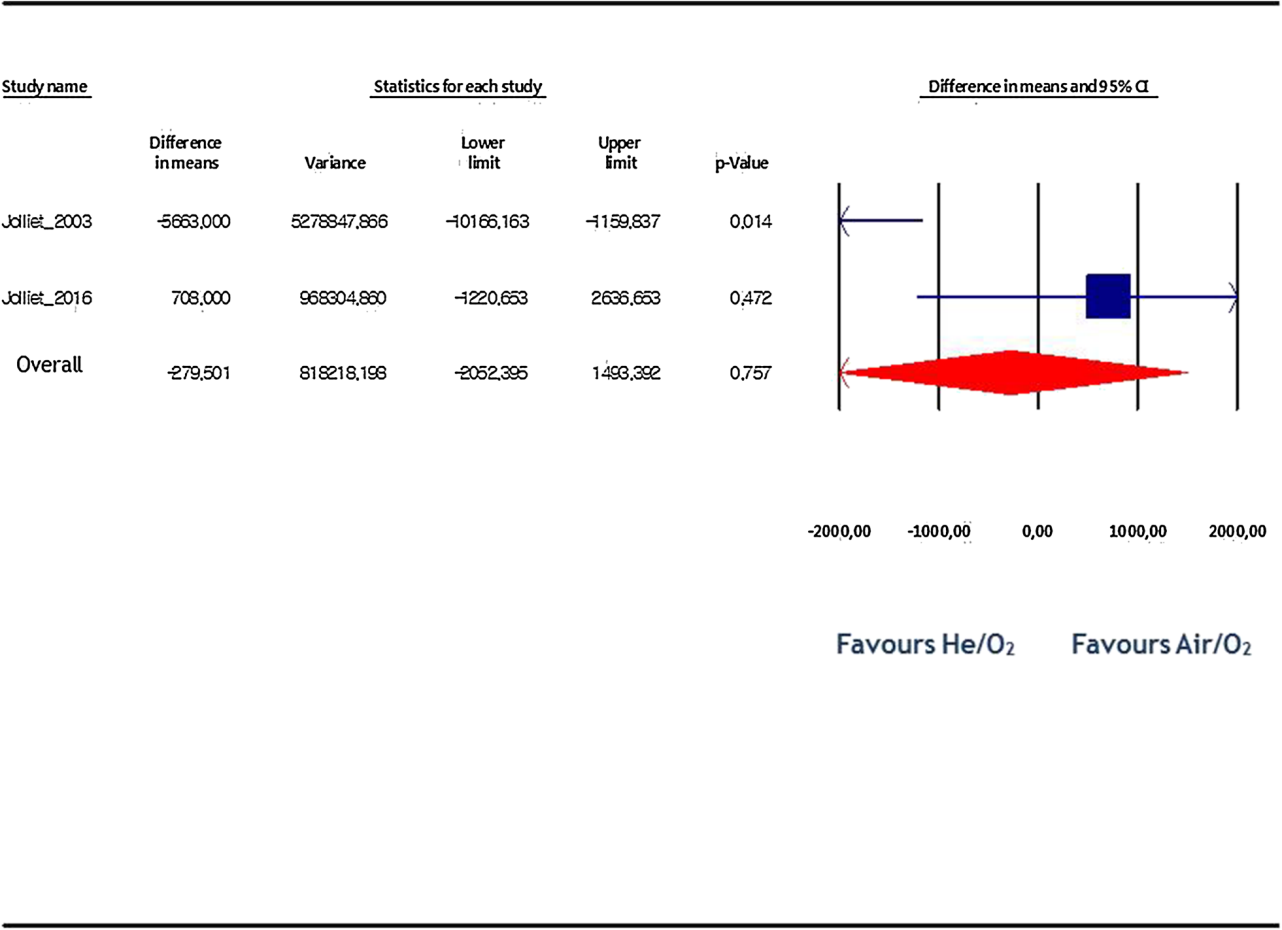
Difference in total costs (per patient) of the initial admission. There was no statistical difference between study groups with a high heterogeneity level between studies (I^2^: 85%)

 The length of ICU stay was also significantly lower in the He/O_2_ group compared to the standard treatment group: difference in means = −1.07 day 95% CI −2.14 to –0.004, p = 0.049, *I* ^2^: 0% (Fig. 5). Regarding total hospital costs incurred by hospital stay and NIV gas (air or helium), there was no statistical difference between both study groups: difference in mean = −279$ by fixed-effect model, 95% CI −2052 to 1493, p = 0.76, *I* ^2^: 85% (Fig. 6).

## Discussion

The current meta-analysis of controlled studies evaluating the use of He/O_2_ as a driving gas for NIV in hypercapnic COPD exacerbation found no significant reduction in either the failure rate of NIV or ICU mortality. However, He/O_2_ significantly reduced the length of ICU stay and the rate of NIV-associated complications.

Beyond the lack of statistical heterogeneity in the main or secondary outcomes analysis, one of the strengths of the current meta-analysis is the lack of clinical heterogeneity incurred by the three included studies. Indeed, the included patients were fairly similar between the first and last study with similar levels of baseline FEV1, pH at inclusion, predicted mortality, etc. In addition, given the relevance of the ventilatory strategy to NIV success/failure, the investigators applied a so-called bundle ventilatory strategy that sought to conform both to evolving technological advances (quality of the interface, compatibility of accessories and ventilators with helium gas) and to clinical standards (mode and ventilatory settings, NIV sessions duration, interaction with patients). Moreover, in each new study, researchers tried to tackle shortcomings of the preceding one as reflected by the extension of the duration of administration of the gas to NIV-free periods in the most recent study [19]. The participating research teams also had comparable levels of performance and mastering of NIV techniques, and have clearly benefited from the learning curve of NIV implementation. This observation is reflected by a steady reduction in the failure rate recorded in the three studies, a fact that negatively impacted subsequent study design. Indeed, in the three RCTs that evaluated He/O_2_ in hypercapnic COPD exacerbation, there was a recurrent overestimation of the NIV failure rate in the control group, leading to an underestimation of the sample size, thereby yielding substantially underpowered trials. Yet, the first two RCTs conducted by Jolliet et al. [20] and Maggiore et al. [21] exhibited a reduction in the intubation rate with He/O_2_ which went well beyond what could be considered as a minimal clinically relevant effect. In fact, these studies were conducted at times when the “learning curve” of NIV in real life was still in its ascending limb [22]. For example, Jolliet et al. [20] assumed an intubation rate of 45%, the rate assumed by Maggiore et al. [21] was 40%, while these assumptions were reduced to 25% in the recent and largest study conducted by Jolliet et al. [19]. However, the observed intubation rate recorded in the control group of each study was actually much lower, amounting to 20, 30.4, and 14.5%, respectively [19–21]. Of note, the ECHO ^ICU^ study, which was the largest study on the evaluation of He/O_2_ mixture in hypercapnic COPD exacerbation, recorded the lowest rate of primary outcome event, i.e., NIV failure and tracheal intubation [19].

Should a new study be conducted in order to provide a definitive answer on the benefit of He/O_2_ in hypercapnic COPD exacerbation? Besides the cumbersome logistics (ventilators with helium option, gas cylinders blended with the needed He/O_2_ mixture, specific high-concentration facial masks using He/O_2_), the sample size needed to detect a clinically relevant reduction in the NIV failure rate (considering that recorded with standard air/O_2_, 14.5%) would amount to no less than 1000 patients in each arm, with a type 1 and type 2 errors of 5 and 10%, respectively [19].

We cannot readily account for the observed reduction in the rate of NIV complications by the use of He/O_2_ mixture. Explanation cannot be based solely on differences in the properties of the two inhaled mixtures with the change in the flow pattern from turbulent to laminar. Explanation must remain a matter of speculation at the present time.

The initial assumption of systematically substituting air/O_2_ by He/O_2_ in patients with hypercapnic COPD exacerbation seems actually unreasonable in the light of the downward trend in the failure rates reported in the most recent studies, particularly those issued from well-trained teams [2, 5]. He/O_2_ mixture becomes in this context a much less attractive option given its constraining logistics and high costs. However, real-life surveys have recently reported significantly higher failure rates than those observed by Jolliet et al. in the most recent RCT, where the experienced participating teams and a potential study effect probably had a positive impact. The cost–benefit trade-off could under these conditions still lean toward the use of He/O_2_ [1, 9, 23, 24]. Nonetheless, it seems unrealistic to propose He/O_2_ invariably to all patients with hypercapnic COPD exacerbation [25]. Because the response to He/O_2_ mixture breathing has a large variability between subjects according to diseases phenotypes, every effort should be made to identify a subgroup of patients who might derive a real clinical benefit from the physiological effects of He/O_2_, which may make the small difference that would reduce the intubation rate further [15, 26–29]. Indeed, the use of He/O_2_ instead of air/O_2_ in such patients may reduce the work of breathing and dynamic hyperinflation to a sufficient level to avert intubation. Clinical indicators that are well correlated with the work of breathing, and capable of detecting impending respiratory muscle fatigue, are therefore warranted and need to be validated in the clinical setting [30–32]. Identifying such a subgroup of potential responders to He/O_2_ mixture can also be addressed in a specifically designed RCT, or through an individual patient data meta-analysis.

## Conclusion

in the light of the results uncovered by the current meta-analysis, there is insufficient evidence to recommend the systematic use of He/O_2_ in all patients with hypercapnic COPD exacerbation requiring NIV despite the reduction in the ICU length of stay and NIV side effects, given the constraining logistics. One may argue that with standard air/O_2_ we have probably reached an acceptable level of NIV failure. However, a subset of patients (which remains to be clearly delineated) at high risk of NIV failure might benefit from the use of He/O_2_, and efforts should now be directed at identifying such a subgroup.

## Abbreviations

CI: Confidence interval
COPD: Chronic obstructive pulmonary disease
DRG: Diagnosis-related group
ICU: Intensive care unit
NIV: Noninvasive ventilation
OR: Odds ratio
PRISMA: Preferred Reporting Items for Systematic Reviews and Meta-Analysis
RCT: Randomized controlled trial
VAP: Ventilator-associated pneumonia

## References

[1] Lindenauer PK, Stefan MS, Shieh MS, Pekow PS, Rothberg MB, Hill NS (2015). Hospital patterns of mechanical ventilation for patients with exacerbations of COPD. Ann Am Thorac Soc..

[2] Stefan MS, Nathanson BH, Higgins TL, Steingrub JS, Lagu T, Rothberg MB (2015). Comparative Effectiveness of Noninvasive and Invasive Ventilation in Critically Ill Patients With Acute Exacerbation of Chronic Obstructive Pulmonary Disease. Crit Care Med.

[3] Stefan MS, Shieh MS, Pekow PS, Hill N, Rothberg MB, Lindenauer PK (2015). Trends in mechanical ventilation among patients hospitalized with acute exacerbations of COPD in the United States, 2001 to 2011. Chest.

[4] Lindenauer PK, Stefan MS, Shieh MS, Pekow PS, Rothberg MB, Hill NS (2014). Outcomes associated with invasive and noninvasive ventilation among patients hospitalized with exacerbations of chronic obstructive pulmonary disease. JAMA Intern Med..

[5] Ouanes I, Ouanes-Besbes L, Ben Abdallah S, Dachraoui F, Abroug F (2015). Trends in use and impact on outcome of empiric antibiotic therapy and non-invasive ventilation in COPD patients with acute exacerbation. Ann Intensive Care..

[6] Girou E, Brun-Buisson C, Taille S, Lemaire F, Brochard L (2003). Secular trends in nosocomial infections and mortality associated with noninvasive ventilation in patients with exacerbation of COPD and pulmonary edema. JAMA.

[7] Girou E, Schortgen F, Delclaux C, Brun-Buisson C, Blot F, Lefort Y (2000). Association of noninvasive ventilation with nosocomial infections and survival in critically ill patients. JAMA.

[8] Brochard L, Mancebo J, Wysocki M, Lofaso F, Conti G, Rauss A (1995). Noninvasive ventilation for acute exacerbations of chronic obstructive pulmonary disease. N Engl J Med.

[9] Chandra D, Stamm JA, Taylor B, Ramos RM, Satterwhite L, Krishnan JA (2012). Outcomes of noninvasive ventilation for acute exacerbations of chronic obstructive pulmonary disease in the United States, 1998-2008. Am J Respir Crit Care Med.

[10] Jolliet P, Tassaux D, Thouret JM, Chevrolet JC (1999). Beneficial effects of helium:oxygen versus air:oxygen noninvasive pressure support in patients with decompensated chronic obstructive pulmonary disease. Crit Care Med.

[11] Morice AH (2000). Helium/oxygen and severe COPD. Lancet.

[12] Laden G (2001). Helium/oxygen and severe COPD. Lancet.

[13] Allan PF, Thomas KV, Ward MR, Harris AD, Naworol GA, Ward JA (2009). Feasibility study of noninvasive ventilation with helium-oxygen gas flow for chronic obstructive pulmonary disease during exercise. Respir Care.

[14] Hussain O, Collins EG, Adiguzel N, Langbein WE, Tobin MJ, Laghi F (2011). Contrasting pressure-support ventilation and helium-oxygen during exercise in severe COPD. Respir Med.

[15] Jaber S, Fodil R, Carlucci A, Boussarsar M, Pigeot J, Lemaire F (2000). Noninvasive ventilation with helium-oxygen in acute exacerbations of chronic obstructive pulmonary disease. Am J Respir Crit Care Med.

[16] Pecchiari M, Pelucchi A, D’Angelo E, Foresi A, Milic-Emili J, D’Angelo E (2004). Effect of heliox breathing on dynamic hyperinflation in COPD patients. Chest.

[17] Tassaux D, Gainnier M, Battisti A, Jolliet P (2005). Helium-oxygen decreases inspiratory effort and work of breathing during pressure support in intubated patients with chronic obstructive pulmonary disease. Intensive Care Med.

[18] Higgins JPT AD, Sterne JAC, editors. Chapter 8: Assessing risk of bias in included studies. Cochrane Handbook for Systematic Reviews of Interventions Version 510: Cochrane 2011; 2011.

[19] Jolliet P, Ouanes-Besbes L, Abroug F, Ben Khelil J, Besbes M, Garnero A, et al. A Multicenter Randomized Trial Assessing the Efficacy of Helium/Oxygen in Severe Exacerbations of Chronic Obstructive Pulmonary Disease. American journal of respiratory and critical care medicine. 2016. 10.1164/rccm.201601-0083OC 27736154

[20] Jolliet P, Tassaux D, Roeseler J, Burdet L, Broccard A, D’Hoore W (2003). Helium-oxygen versus air-oxygen noninvasive pressure support in decompensated chronic obstructive disease: a prospective, multicenter study. Crit Care Med.

[21] Maggiore SM, Richard JC, Abroug F, Diehl JL, Antonelli M, Sauder P (2010). A multicenter, randomized trial of noninvasive ventilation with helium-oxygen mixture in exacerbations of chronic obstructive lung disease. Crit Care Med.

[22] Schnell D, Timsit JF, Darmon M, Vesin A, Goldgran-Toledano D, Dumenil AS (2014). Noninvasive mechanical ventilation in acute respiratory failure: trends in use and outcomes. Intensive Care Med.

[23] Gacouin A, Jouneau S, Letheulle J, Kerjouan M, Bouju P, Fillatre P (2015). Trends in Prevalence and Prognosis in Subjects With Acute Chronic Respiratory Failure Treated With Noninvasive and/or Invasive Ventilation. Respiratory care..

[24] Toft-Petersen AP, Torp-Pedersen C, Weinreich UM, Rasmussen BS (2017). Trends in assisted ventilation and outcome for obstructive pulmonary disease exacerbations. A nationwide study. PloS one..

[25] Carr J, Jung B, Chanques G (2012). Jaber S.

[26] Mutlu GM, Budinger GRS (2010). Not much turbulence: addition of heliox to noninvasive ventilation fails to improve outcomes in patients with exacerbations of chronic obstructive pulmonary disease. Crit Care Med.

[27] Burgel PR, Paillasseur JL, Peene B, Dusser D, Roche N, Coolen J (2012). Two distinct chronic obstructive pulmonary disease (COPD) phenotypes are associated with high risk of mortality. PLoS ONE.

[28] Adler D, Pepin JL, Dupuis-Lozeron E, Espa-Cervena K, Merlet-Violet R, Muller H, et al. Comorbidities and Subgroups of Patients Surviving Severe Acute Hypercapnic Respiratory Failure in the ICU. American journal of respiratory and critical care medicine. 2016. 10.1164/rccm.201608-1666OC 27973930

[29] Turner AM, Tamasi L, Schleich F, Hoxha M, Horvath I, Louis R (2015). Clinically relevant subgroups in COPD and asthma. European respiratory review: an official journal of the European Respiratory Society..

[30] Roche N, Chavaillon JM, Maurer C, Zureik M, Piquet J (2014). A clinical in-hospital prognostic score for acute exacerbations of COPD. Respir Res.

[31] Quintana JM, Esteban C, Unzurrunzaga A, Garcia-Gutierrez S, Gonzalez N, Barrio I (2014). Predictive score for mortality in patients with COPD exacerbations attending hospital emergency departments. BMC Med.

[32] Contou D, Fragnoli C, Córdoba-Izquierdo A, Boissier F, Brun-Buisson C, Thille AW (2013). Noninvasive ventilation for acute hypercapnic respiratory failure: intubation rate in an experienced unit. Respiratory care..

